# Prolactinoma: Clinical Characteristics, Management and Outcome

**DOI:** 10.7759/cureus.29822

**Published:** 2022-10-02

**Authors:** Hira Irfan, Waqas Shafiq, Ahmed Imran Siddiqi, Sara Ashfaq, Sadaf Attaullah, Asim Munir Alvi, Sardar Ali Khan, Muhammad Abu Bakar, Umal Azmat

**Affiliations:** 1 Endocrinology and Diabetes, Shaukat Khanum Memorial Cancer Hospital and Research Centre, Lahore, PAK; 2 Endocrinology, Shaukat Khanum Memorial Cancer Hospital and Research Centre, Lahore, PAK; 3 Biostatistics and Epidemiology, Shaukat Khanum Memorial Cancer Hospital and Research Centre, Lahore, PAK

**Keywords:** bromocriptine, cabergoline, giant prolactinoma, macroadenoma, microadenoma

## Abstract

Aim

Prolactinoma, a prolactin (PRL) secreting functioning pituitary tumor, is the most common of all pituitary adenomas (PA) accounting for 40-60% and dopamine agonists (DA) are the cornerstone of treatment. The aim of this study was to review the clinical presentation, treatment modalities and therapeutic outcomes of patients with prolactinomas in the South Asia region.

Methods

This retrospective study was conducted in the Endocrinology Department of Shaukat Khanum Memorial Cancer Hospital and Research Centre from December 2011 till December 2019. Medical records were used to retrieve for patient’s demographics, clinical features at diagnosis, PRL levels and size of prolactinoma on MRI at diagnosis and after start of dopamine agonists and outcome of medical management.

Results

A total of 107 patients were included in this study. Mean age at diagnosis was 35 (22-54) years for men and 32 (18-50) years for women and 66.4% (71) of the patients were females. Our study included 38 (35.5%) microadenoma, 50 (46.7%) macroadenoma and 19 (17.8%) giant adenomas. At presentation, the most common symptom among females was menstrual irregularity/amenorrhea seen in 73.2% of females and among males was visual disturbance (80.6%). A significant reduction in PRL levels was seen within six to 12 months of treatment. Mean PRL levels decreased from 3162.8 ng/ml to 1.52 ng/ml. A notable decrease in tumor size was seen with medical management, mean adenoma size decreased from 2.18 cm to 1.04 cm. With cabergoline (CAB) 83.3% biochemical cure was seen compared to bromocriptine (BRC) which has 60.4%. The radiological response rate in CAB and BRC groups was 65.45% and 60%, respectively. Complete resolution of adenoma was seen in 13 patients (nine were microadenoma, two macro and two giant adenomas). The prolactin level at diagnosis was positively correlated with maximum tumor diameter (r = 0.469, P = 0.001). Two patients developed cerebrospinal fluid (CSF) rhinorrhea and the defect was repaired in both patients. Median follow-up duration was 40 (12-288) months.

Conclusion

Clinical presentation and demographics of prolactinoma are the same in our region when compared to the rest of the world. Cabergoline is superior to bromocriptine in prolactin normalization and tumor shrinkage but still bromocriptine is being used in a significant number of patients in low-income countries as first-line due to its low cost.

## Introduction

Prolactinoma, a prolactin-secreting functioning pituitary tumor, is the most common of all pituitary adenomas (PA) accounting for 40-60% and up to 80% if microadenomas (size less than 1 cm) are included [1]. The mean prevalence is approximately 10 per 100,000 in men and 30 per 100,000 in women, with peak prevalence for women aged 25-34 years [2]. Most of the clinical features of prolactinoma are due to overproduction of prolactin, causing hormonal imbalance leading to menstrual abnormalities and galactorrhea in females, and in men it can cause erectile dysfunction, diminished libido, and infertility. Neurological manifestations in form of psychosis, anxiety, and sleep disturbances are found in both men and women. In addition, macroadenomas (size greater than 1 cm) and giant prolactinoma (size greater than 4 cm) may also cause mass effects leading to headache and visual disturbances. The term giant prolactinoma is coined in literature to demarcate prolactinomas that are extremely large and greater than 4 cm in diameter [3]. Females seek medical advice earlier due to prolactin causing inhibition of gonadotropins leading to menstrual irregularities, galactorrhea, and infertility. On the contrary, males seek medical attention late due to symptoms of mass effect and less specific symptoms of hypogonadism.

For management purposes, it is of paramount importance to differentiate prolactinoma from nonfunctioning pituitary adenoma causing hyperprolactinemia. Prolactin level should be raised to a significant value for the diagnosis of prolactinoma. Prolactin levels are generally proportional to tumor size and a level greater than 94 ng/ml can differentiate prolactinoma from nonfunctioning pituitary adenoma [4]. Magnetic resonance imaging (MRI) with pituitary protocol is the gold standard protocol to look for the size and dimensions of adenoma and invasion of surrounding structures. Follow-up MRI is recommended to look for tumor shrinkage and resolution of adenoma after initiation of therapy. First-line management of prolactinoma is with dopamine agonists (DA) and studies have shown that cabergoline (CAB) is more effective in lowering prolactin (PRL) levels, reducing tumor size, and has less adverse effects when compared to bromocriptine [5]. Surgery and/or radiotherapy are options which can be used when a prolactinoma is causing significant mass effect, especially on the optic chiasma (threatening blindness), or if there is intolerance or resistance to medical therapy. Trans-sphenoidal pituitary surgery being minimally invasive and having relatively good safety profile is favored among other approaches [6,7].

Careful clinical and biochemical follow-up is recommended for patients with prolactinoma. Therapies may be tapered or discontinued in patients who received a minimum of two years of DA and no longer have elevated serum prolactin levels and no visible tumor remnant on MRI [4]. Clinical data with regards to baseline characteristics, clinical features, management and outcome in prolactinoma patients has been extensively studied in the western population. However, in our population due to prolactinoma being a relatively less commonly diagnosed entity, there is a paucity of data. To our knowledge, only one study has been conducted in past on characteristics of prolactinoma in our region, but it has a small sample size and a short duration of follow-up [8]. The aim of our study is to provide local data which can be used as a reference for characteristics, clinical presentation management and outcome of prolactinomas in developing countries. In addition, we also aspire to look for challenges low-income countries face while providing state-of-the-art treatment comparable to international standards.

## Materials and methods

This retrospective study was conducted at the Department of Endocrinology at Shaukat Khanum Memorial Cancer Hospital and Research Centre (SKMCH & RC). Record of prolactinoma patients from December 2011 to December 2019 was extracted from the hospital database. The institutional review board (IRB; study IRB# EX-15-05-20-02) of SKMCH & RC approved the current retrospective study. IRB-SKMCH & RC allowed the waiver for informed consent for this study since the data was accessed through the hospital information system (HIS) and was restricted to the researcher group only and patients were allocated serial numbers to conceal patient identity.

Adult patients with radiological evidence of pituitary adenoma with prolactin greater than 94 ng/ml were included in the study. Patients with other causes of hyperprolactinemia such as pituitary stalk compression, co-secretory pituitary adenomas, infiltrative diseases like sarcoidosis and tuberculosis, certain medications and pregnancy were excluded.

Prolactin was measured by an immunochemiluminescent assay (ICMA) (IMMULITE 2000) with a sensitivity of 0.5 ng/ml. Reference levels for men in our laboratory are 2.50-17 ng/ml and for women are 1.90-25 ng/ml. Levels greater than 150 ng/ml were calculated after appropriate serial dilutions for any possible hook effect. Pituitary adenomas were evaluated radiologically by MRI and maximum tumor diameter was recorded. Based on tumor diameter adenomas were classified as microadenoma (<1 cm), macroadenoma (≥1 cm) and giant prolactinoma (≥4 cm).

A total of 107 patients were identified and included in the study through retrospective analysis of hospital records. Treatment history with either CAB or bromocriptine (BRC) was noted. Treatment with more than one DA may have occurred due to drug intolerance, drug resistance, drug unavailability or a combination of these factors. The outcome was calculated in terms of biochemical, radiological, and clinical response. Biochemical response is normalization of prolactin level (<25 ng/ml). Radiological response is documented 50% or more reduction in tumor size or resolution of adenoma on follow-up MRI. Clinical response is improvement or resolution of clinical features/visual disturbance with dopamine agonists.

The following variables were retrieved from the hospital electronic record for analysis: age, gender, clinical presentation including presenting complaint and examination findings if any, size of adenoma, DA used, outcome of medical management and mean duration of follow-up. Pretreatment prolactin level (baseline) and then at 3, 6, 12, 18, 24, 36 and 60 months after starting medical management was noted. The size of adenoma was recorded on MRI done at diagnosis (baseline) and later at 6, 12, 24, 36 and 60 months.

Statistical analysis was carried out using the SPSS software version 20.0 (IBM Corp., Armonk, NY, USA). Continuous variables were stated as Median and categorical variables were computed as frequencies and percentages. Categorical variables were compared using the chi-square test or Fisher’s exact test (when necessary). The continuous variables were compared using the independent t-test. One-way ANOVA was used to check the mean difference among three different diagnoses. Statistical significance was defined as a two-tailed P-value of 0.05.

## Results

In our study population, the majority of the patients were female (71; 66.4%). The mean age at diagnosis was 35 (22-54) years for men and 32 (18-50) years for women. Baseline median prolactin level at presentation was 510 ng/ml (61-56670) and mean adenoma size was 2.18 cm (0.20-7.0). Out of 107 pituitary tumors, 55 had extrasellar extension. A total of 72% macroadenomas and all giant prolactinomas had extrasellar extension. Optic chiasm involvement or mass effect was seen in 44 patients whereas 40 patients had extension into cavernous sinus. The rest of the baseline characteristics are as shown in Table 1.

**Table 1:** Clinical Characteristics

N (%) 107	
Age (years)	
Male (Mean)	35 (22-54)
Female (Mean)	32 (18-50)
Gender	
Male	36 (33.6)
Female	71 (66.4)
Medication	
Bromocriptine	34 (31.8)
Cabergoline	59 (55.1)
Both drugs	14 (13.08)
Causes of increased prolactin	
Microadenoma	38 (35.5)
Macroadenoma	50 (46.7)
Giant prolactinoma	19 (17.8)
Extrasellar extension	55
Microadenoma	0
Macroadenoma	36 (72)
Giant prolactinoma	19 (100)
Pretreatment prolactin level	
Median (minimum-maximum)	510 (61-56670)
Pretreatment tumor size (cm)	
Mean± SD, Range	2.18±1.70, (0.20-7.0)
Posttreatment tumor size (cm)	
Mean±SD, Range	1.04 ± 0.99 (0.00-4.50)
Follow-up (months)	
Median (minimum-maximum)	40 (12-288)

The common symptoms at presentation in female subjects were menstrual irregularities, headache, and galactorrhea in 73.2%, 47.9% and 42.3%, respectively. Whereas in males, visual disturbance (80.6%) followed by headache (75.0%) and erectile dysfunction (30.6%) were the commonly reported complaints as depicted in Table 2.

**Table 2:** Gender-based representation of clinical features

	Male 36 (33.6%)	Female 71 (66.4%)	p-value
Visual disturbance			0.001
43 (40.1)	29 (80.6)	14 (19.7)	
Headache			0.01
61 (57)	27 (75.0)	34 (47.9)	
Incidental			0.40
6 (5.6)	3 (8.3)	3 (4.2)	
Erectile dysfunction			0.001
11 (10.3)	11 (30.6)	-	
Menstrual irregularities			0.001
52 (48.6)	-	52 (73.2)	
Galactorrhea			0.001
30 (28)	-	30 (42.3)	
Infertility			0.15
20 (18.7)	4 (11.1)	16 (22.5)	
Behavioral disturbance			0.54
11 (10.3)	5 (13.9)	6 (8.5)	
Gynecomastia			0.54
1 (0.9)	1 (2.8)	-	
Seizures			0.50
1 (0.9)	-	1 (1.4)	
Weight gain			0.54
1 (0.9)	-	1 (1.4)	
Causes of increased prolactin			0.001
Microadenoma	3 (8.3)	35 (49.3)	
Macroadenoma	18 (50.0)	32 (45.1)	
Giant prolactinoma	15 (41.7)	4 (5.6)	

The overall prolactin (PRL) level at diagnosis was positively correlated with maximum tumor diameter (r = 0.469, P = 0.001). Cabergoline was given to 59 (55.1%) patients, bromocriptine to 34 (31.8%) patients and 14 (13.08%) patients received both DA at some point during treatment. Bromocriptine was later changed to cabergoline attributable to drug side effects in eight patients and drug resistance in five patients. Cabergoline had to be switched to bromocriptine due to non-affordability in one patient.

Biochemical response to cabergoline and bromocriptine was assessed. A significant prolactin reduction was seen within 6-12 months of treatment with DA (both cabergoline and bromocriptine). Mean prolactin level decreased from 3162.8 ng/ml to 1.52 ng/ml (p-value = 0.01). Within two years, 73.2% of cases had prolactin level normalization and the majority had received cabergoline, as shown in Table 3 with a p-value of 0.03.

**Table 3:** Biochemical outcome

	Not Achieved 26 (26.8%)	Achieved 71 (73.2%)	p-value
Medications			0.03
Bromocriptine	11 (42.3)	19 (26.8)	
Cabergoline	9 (34.6)	45 (63.4)	
Both	6 (23.1)	7 (9.9)	
Causes of increased prolactin			0.002
Microadenoma	3 (11.5)	33 (46.5)	
Macroadenoma	15 (57.7)	29 (40.8)	
Giant prolactinoma	8 (30.8)	9 (12.7)	

**Figure 1:**
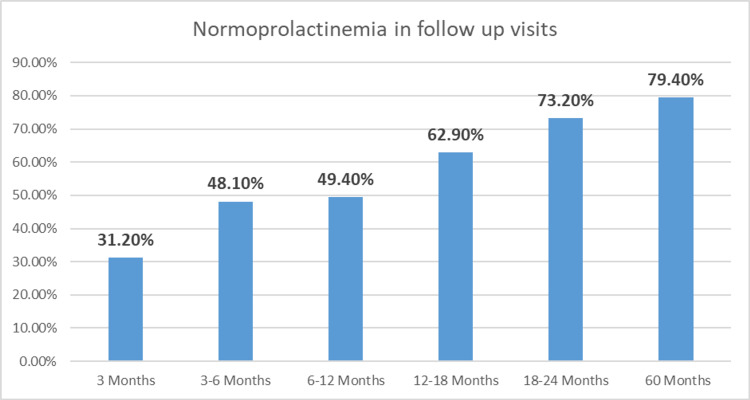
Normoprolactinemia in follow-up visits. Data is shown in percentages.

Forty-five (83.3%) out of 54 patients who received cabergoline and 19 (63.3%) out of 30 patients taking bromocriptine had normalization of prolactin within two years. In the remaining 10 patients, nine were lost to follow-up after the first clinic visit and one patient had not checked prolactin. At the last documented follow-up visit, 79.4% of patients achieved biochemical outcome with DA, either cabergoline or bromocriptine or both as shown in Figure 1.

Prolactin normalization with medical management was significantly higher in microadenoma as compared to in macroadenoma and giant prolactinoma (88.9% vs 65.9% and 52.9% respectively; p-value = 0.002).

Significant shrinkage in adenoma was noted post-treatment. Mean tumor size at the time of diagnosis decreased from 2.18 cm to 1.04 cm measured on the last follow-up (Table 1). At two years follow-up visit, 96 patients underwent MRI, amongst them 44 (45.8%) had more than 50% reduction in tumor size. MRI of 98 patients at the last documented follow-up visit was recorded. Response increased to 62.2% at the last documented visit from 45.8% (at two years). Out of 98 patients, 55 received cabergoline, 30 received BRC and 13 received both drugs but at separate times. Radiologic outcome was achieved in 36 (65.45%) cases receiving CAB and 18 (60%) receiving BRC. Subgroup analysis is as shown in Table 4.

**Table 4:** Radiological outcome

	No Reduction 37 (37.8%)	Reduction 61 (62.2%)	p-value
Medications			0.71
Bromocriptine	12 (32.4)	18 (29.5)	
Cabergoline	19 (51.4)	36 (59.0)	
Both	6 (16.2)	7 (11.5)	
Causes of increased prolactin			0.72
Microadenoma	12 (32.4)	24 (39.3)	
Macroadenoma	17 (45.9)	27 (44.3)	
Giant prolactinoma	8 (21.6)	10 (16.4)	

More than 50% reduction in tumor size was seen in 24 (60.6%) out of 36 microadenoma, 27 (61.4%) out of 44 macroadenoma and 10 (55.6%) out of 18 giant prolactinomas. Patients who were followed for more than two years showed a greater radiological response. Thirty-one patients were followed for more than five years and radiological outcome was achieved in 70% of patients.

Complete resolution of adenoma was seen in 13 patients (nine were microadenoma, two macro and two giant adenomas). Amongst them, 11 had received cabergoline and two had received bromocriptine. Two patients had progressive disease (one giant prolactinoma and one macroadenoma). After starting DA two patients developed cerebrospinal fluid (CSF) rhinorrhea (one giant and one macroadenoma). Both patients presented within one month of starting bromocriptine. A significant reduction of prolactin level and size of tumor was seen after repair in one patient. Treatment response was not assessed in the second patient because he was lost to follow up after the repair of defect.

Clinical response was documented as either improvement in symptoms or visual defect or resolution of clinical symptoms or visual defect. Most patients (74.5%; 73/98) showed improvement or resolution of clinical symptoms in 6-12 months of treatment with DA, and it was recorded at the last follow-up that 91 (92.9%) patients reported improvement or resolution of symptoms as shown in Table 5. During clinical encounters, among 45 patients who had visual disturbance 23 (51.1%) had significant improvement three (6.7%) had complete resolution and 15 (33.34%) had persistent visual field defects with medical management.

**Table 5:** Clinical Outcome

	Not achieved 7 (7.1%)	Achieved 91 (92.9%)	p-value
Medications			0.30
Bromocriptine	4 (13.3)	26 (86.70)	
Cabergoline	2 (3.6)	53 (96.4)	
Both	1 (7.7)	12 (92.3)	
Causes of increased prolactin			0.03
Microadenoma	2 (5.6)	34 (94.4)	
Macroadenoma	1 (2.3)	43 (97.7)	
Giant prolactinoma	7 (7.1)	14 (77.8)	

DA withdrawal was attempted in eight cases. Three patients had successful withdrawal of the drug and are under surveillance. The drug was restarted in five patients due to recurrence of symptoms and an increase in prolactin levels within 6-12 months of tapering. Eight cases of macroadenoma were kept at the lowest possible dose of DA and there was no recurrence of tumor on the lower suppressive dose.

## Discussion

So far there is well-established data in the literature suggesting that medical management with DA is the mainstay of treatment of prolactinoma [9,10]. Compared to the international literature on prolactinoma, there is a paucity of data in our region. To our knowledge, only one study was conducted in our country to analyze the outcome of medical management in prolactinoma but with a small sample size (n=68) [8]. In recent years cabergoline has gained popularity over bromocriptine in the treatment of prolactinoma because cabergoline controls prolactinoma more efficiently and is better tolerated [4]. In one meta-analysis of four randomized trials by Santos et al. (sample size of 743 patients) [11], normalization of serum PRL levels showed a significant difference in favor of the cabergoline group (RR 0.67 [CI 95% 0.57, 0.80]) and adverse events such as nausea and vomiting were significantly less frequent in the cabergoline-treated patients, (RR 1.66 [CI 95% 1.33, 2.06]) and (RR 2.02 [CI 95% 1.13, 3.59], respectively). In our low-income and developing region bromocriptine is still being widely used due to its low cost and accessibility. In our study, we observed that a significant number of patients were started on bromocriptine instead of cabergoline due to its affordability. In our study, 55.1% of patients received cabergoline and 31.8% received bromocriptine.

We compared the clinical characteristics of men and women at presentation and found a similar trend in our region. In one retrospective cohort study [12], prolactinoma was the most common pituitary adenoma accounting for 57.4% of all adenomas, with female gender predominance (81%) and female patients being younger. Similarly, in our study, the majority of our population was female (66.4%) and had earlier presentation than men (mean age was 35 years for men and 32 years for women). While comparing symptoms at presentation of prolactinoma, our results were comparable to international data [10,13]. In a single center experience of 12 years by Almalki et al. [13], it was observed that the majority of patients presented with headache (87.8%), in males most common clinical presentation was visual disturbance (70.8%) and among females, it was amenorrhea (55.6%). We also found that headache is one of the most common symptoms among all prolactinoma patients and the majority of females presented with menstrual irregularities (73.2%, p-value <0.001). The most common presenting symptom among males was visual disturbance (80.6% with a p-value of 0.001) and 30.6% of patients presented with erectile dysfunction.

It has been suggested in the literature that prolactinomas in men are larger than in females. In a study conducted by Delgrange et al. [14], of 45 men and 51 women with prolactinomas, prolactinomas were significantly larger in men of all ages. This is likely due to the later presentation of men. We observed a similar trend in our study with giant prolactinoma more prevalent in males (15 of 19; P <0.001) and microadenoma being more common in females (35 of 38; P <0.001). In our cohort, we calculated a positive correlation between prolactin level and tumor diameter (r = 0.469, P = 0.001).

A review by Duskin-Bitan and Shimon [15], summarizing 15 cohorts of patients with prolactinoma treated medically mostly with cabergoline, showed 76% of men normalized prolactin during follow-up. Our study also showed that a considerable number of patients showed normalization of prolactin within two years of treatment (73.2%).

This study clearly shows that cabergoline (CAB) is superior to bromocriptine (BRC) in achieving the outcome which is comparable to previously published data. In a recent article by Rudman et al. [16], macroprolactinomas in men were controlled with cabergoline in 84% of cases. In another retrospective comparison of CAB and BCR in hyperprolactinemia by Arduc et al. [17], CAB was found more effective than BCR in controlling symptoms of hormone excess, normalization of PRL (87.4 vs. 41.4%) and tumor shrinkage (79.8 ± 39.1 vs. 54.1 ± 55.3%). We also found an overall similar response. We observed 83.3% biochemical response with cabergoline and 63.3% with bromocriptine suggesting that cabergoline is more effective than bromocriptine in controlling prolactin level. The radiological response rate in our study was seen in 65.45% treated with CAB and 60% treated with BCR documented at the last follow-up visit.

In one review article [18], summarizing 12 published series of patients with macroprolactinomas (n=309) treated with CAB, prolactin normalized in 80% of the cases and the tumor shrunk significantly in 87% of patients. The reason for the comparatively low radiological response in our study (65.45%) compared to this study is probably because we still use bromocriptine in a significant number of patients which is inferior to cabergoline in tumor shrinkage. Another reason is that we calculated a 50% or more reduction in tumor volume, whereas other studies in this article included any shrinkage in lesion.

In a cohort of 71 patients of giant prolactinoma by Hamidi et al., 55% of patients had PRL normalization and 26% had no visible tumor at follow-up [19]. In our cohort, we observed a similar trend in prolactin normalization (52.9% giant prolactinomas showed prolactin normalization) and complete resolution of the tumor was seen in only two giant prolactinomas (10.5%).

DAs are not only effective in prolactin normalization and tumor shrinkage, but patients show remarkable clinical recovery as well. In one single-center retrospective study [20], CAB resulted in improvement in visual field defects in 68% and hypogonadotropic hypogonadism recovered in 32% of the patients after CAB therapy. We observed that 96.4% of subjects with CAB and 86.70% with BCR showed a clinical response and 51.1% of patients had improvement of visual field defects. Relatively, a low percentage of improvement in the visual field in our population may be attributable to the use of bromocriptine in some patients whereas in this international study they only used cabergoline. We also reported two cases of CSF rhinorrhea in our population after starting dopamine agonists. Both required surgical repair of the defect and presented within one month of starting treatment [21].

In our cohort, a few confounders existed. Lack of response in a few patients was attributed to noncompliance with medications and fewer follow-up visits of patients in our region rather than the drug. Also, the cost of medication and laboratory/radiological investigations also led to infrequent investigations and hence follow-up. The exact treatment duration required for achieving outcome could not be assessed in some patients as being a tertiary care setup most patients were already taking DA before presenting to our center.

Further comprehensive analysis is required to look for outcome of other modalities in medically resistant and aggressive prolactinoma, i.e., the use of temozolomide [4,22] and tyrosine kinase inhibitors. Surgical resection of prolactinoma is used as adjunctive treatment if no response to DA is seen. In the era of endoscopic surgery and the development of surgical techniques, it is time to reassess the relationship between DAs and surgery. Internationally multiple retrospective studies and meta-analyses have been conducted to look for the safety and efficacy of surgery as a first-line treatment in prolactinoma [23-25]. This needs to be looked at in our region as well.

## Conclusions

Clinical presentation and demographics of prolactinoma are the same in our region when compared to the rest of the world. Bromocriptine being cost-effective is still being widely used in our region and we found it inferior to cabergoline in prolactin normalization, adenoma shrinkage and improvement of symptoms. Our study reinforces that cabergoline should be used as first line or bromocriptine should be switched to cabergoline if a desirable treatment response is not achieved. Patients should be followed up at regular interval even after stopping dopamine agonists to look for recurrence. The outcome can be improved by insisting on drug compliance.
